# Identification and Validation of a Prognostic Gene Signature for Diffuse Large B-Cell Lymphoma Based on Tumor Microenvironment-Related Genes

**DOI:** 10.3389/fonc.2021.614211

**Published:** 2021-02-22

**Authors:** Tao Pan, Yizi He, Huan Chen, Junfei Pei, Yajun Li, Ruolan Zeng, Jiliang Xia, Yilang Zuo, Liping Qin, Siwei Chen, Ling Xiao, Hui Zhou

**Affiliations:** ^1^ Department of Lymphoma & Hematology, Hunan Cancer Hospital, The Affiliated Cancer Hospital of Xiangya School of Medicine, Central South University, Changsha, China; ^2^ The Third Xiangya Hospital, Central South University, Changsha, China; ^3^ Department of Gastrointestinal Surgery, First Hospital of Jilin University, Changchun, Jilin, China; ^4^ Hunan Province Key Laboratory of Tumor Cellular & Molecular Pathology, Cancer Research Institute, Hengyang School of Medicine, University of South China, Hengyang, China; ^5^ Department of Histology and Embryology of School of Basic Medical Science, Central South University, Changsha, China

**Keywords:** diffuse large B-cell lymphoma, tumor microenvironment, Gene Expression Omnibus database, signature, prognostic

## Abstract

Diffuse large B-cell lymphoma (DLBCL) is an extremely heterogeneous tumor entity, which makes prognostic prediction challenging. The tumor microenvironment (TME) has a crucial role in fostering and restraining tumor development. Consequently, we performed a systematic investigation of the TME and genetic factors associated with DLBCL to identify prognostic biomarkers for DLBCL. Data for a total of 1,084 DLBCL patients from the Gene Expression Omnibus database were included in this study, and patients were divided into a training group, an internal validation group, and two external validation groups. We calculated the abundance of immune–stromal components of DLBCL and found that they were related to tumor prognosis and progression. Then, differentially expressed genes were obtained based on immune and stromal scores, and prognostic TME‐related genes were further identified using a protein–protein interaction network and univariate Cox regression analysis. These genes were analyzed by the least absolute shrinkage and selection operator Cox regression model to establish a seven-gene signature, comprising *TIMP2*, *QKI*, *LCP2*, *LAMP2*, *ITGAM*, *CSF3R*, and *AAK1*. The signature was shown to have critical prognostic value in the training and validation sets and was also confirmed to be an independent prognostic factor. Subgroup analysis also indicated the robust prognostic ability of the signature. A nomogram integrating the seven-gene signature and components of the International Prognostic Index was shown to have value for prognostic prediction. Gene set enrichment analysis between risk groups demonstrated that immune-related pathways were enriched in the low-risk group. In conclusion, a novel and reliable TME relevant gene signature was proposed and shown to be capable of predicting the survival of DLBCL patients at high risk of poor survival.

## Introduction

Diffuse large B-cell lymphoma (DLBCL) is a heterogeneous tumor entity with a striking degree of genetic and clinical heterogeneity. Although more than half of DLBCL patients may achieve long-term remission, the disease remains a major clinical challenge, with approximately 30% of patients not being cured (1, 2). The heterogeneity of the tumor, in particular, poses a major barrier to understanding the genetic basis of the disease and its response to therapy (3). Therefore, there is an urgent need to identify new individual prognostic and risk-stratified biomarkers.

In recent years, the role of the tumor microenvironment (TME) in tumorigenesis has gradually been discovered (4). Studies have revealed that tumor cells are targets of the immune system in the early stages of tumor development; however, over time, these cells begin to resist the innate immune response and then gradually weaken and adapt to it (5–7). Thus, a better understanding of the interactions between the TME and the immune response may provide new approaches to improve the efficiency of current immunotherapies, especially immune checkpoint inhibitor and chimeric antigen receptor (CAR) T cell therapies (8). Several studies have considered the latent role of the TME in the occurrence and development of DLBCL, but their results were controversial (9).

In the era of rituximab and immunotherapy, the ability of the International Prognostic Index (IPI) to predict the prognosis of individual DLBCL patients has decreased (10). A better understanding of the interactions between the TME and IPI scores may provide new approaches to improve response rates to current treatment strategies. Thus, incorporating a prognostic factor from the TME into the existing IPI system would help greatly in the development of prognostic stratification of DLBCL. Here, we propose a compound prognostic nomogram combining a TME-related prognostic model with clinical features. This approach provides a basis for better understanding the molecular mechanisms underlying the prognoses of DLBCL patients.

## Materials and Methods

### Data Sources

Gene expression profiling and clinical data of patients with DLBCL were obtained from the Gene Expression Omnibus (GEO) database (https://www.ncbi.nlm.nih.gov/geo/). Data series were downloaded in a normalized expression matrix file format and were used directly for the analyses. Patients in the GSE31312 dataset were randomly divided into a training group (N = 282) and an internal validation group (N = 188) in a 6:4 ratio. In addition, two DLBCL microarray datasets were used for validation, including 414 DLBCL patients from the GSE10846 dataset and 200 DLBCL patients from the GSE11318 dataset.

### Generation and Analysis of ImmuneScore, StromalScore, and ESTIMATEScore

The ESTIMATE (Estimation of STromal and Immune cells in MAlignant Tumors using Expression data) package in R version 3.6.3 was used to evaluate the abundance of immune and stromal components of the TME from expression data (11). We obtained three scores, ImmuneScore, StromalScore, and ESTIMATEScore, which respectively represent the immune abundance, the stromal abundance, and the sum of both in the TME; for instance, a higher ImmuneScore means a higher abundance of immune components in the TME.

### Identification of Differentially Expressed Genes

To obtain DEGs between high- and low-scoring samples, microarray data from GEO were analyzed using NCBI GEO2R (https://www.ncbi.nlm.nih.gov/geo/geo2r/). 470 patients with GSE31312 were divided by the median of ImmuneScore (StromalScore), and the high and low ImmuneScore (StromalScore) groups were obtained, respectively. Based on comparisons of the high- and low-scoring groups, Adj. *P <*0.05 and fold change >1.05 were set as the thresholds for DEG identification to obtain differential immune and stromal genes. We then took the intersection of immune differential genes and stromal differential genes. The DEGs were visualized using heatmaps (https://software.broadinstitute.org/morpheus/) and Venn plots (http://bioinformatics.psb.ugent.be/webtools/Venn/).

### Functional Enrichment Analyses

DAVID (https://david.ncifcrf.gov/summary.jsp), an online tool for gene functional enrichment, was used for gene ontology (GO) analysis (with respect to cellular component, molecular function, and biological process) and Kyoto Encyclopedia of Genes and Genomes (KEGG) pathway analysis of the 183 DEGs shared between the ImmuneScore groups and the StromalScore groups. The results were displayed using the ggplot2 R package. *P <*0.05 was considered statistically significant.

Gene set enrichment analysis (GSEA) was performed using the gsea-4.0.3 software downloaded from https://www.gsea-msigdb.org/gsea/index.jsp to explore whether immune pathways were significantly different between the high-risk and low-risk groups.

### Protein–Protein Interaction Network Construction and Univariate Cox Regression Analysis

The PPI network was obtained from the STRING database (https://string-db.org/) and reconstructed with version 3.7.2 of Cytoscape. Univariate Cox analysis of overall survival (OS) was used to determine the relationships between expression of 183 DEGs and prognosis. In order to obtain the most critical and meaningful genes to construct the model, we used a method from Bi et al. (12) to further screen the genes. In the PPI network, we kept the core gene located in the center by eliminating genes with fewer peripheral nodes. In univariate Cox regression, the smaller the *p*-value, the more significant the prognosis. Therefore, the sharing factors between the degree of the nodes ≥3 in PPI and the *p <*0.0001 of univariate Cox regression analysis were carried out.

### Generation of the Risk Prediction Model

The training dataset GSE31312 was used to establish the TME risk model. The R package “glmnet” was used for least absolute shrinkage and selection operator (LASSO) Cox regression analysis. A risk formula for predicting prognosis was established by LASSO Cox regression analysis. Then, we calculated the individualized risk score of each patient by dividing all patients into high-risk and low-risk groups using the median risk score as the cut-off value. Kaplan–Meier survival analysis and log-rank test were used to evaluate the difference in OS between the high- and low-risk groups. Time-dependent receiver operating characteristic (ROC) curves were plotted to evaluate prognostic value (13). The analysis was performed using SangerBox (http://sangerbox.com/Tool).

### Univariate and Multivariate Cox Regression for IPI Components and the Seven-Gene Signature

To assess whether the risk prediction model could be used as an independent prognostic indicator for DLBCL patients, univariate and multivariate Cox regression analyses were performed with SPSS 26.

### Construction and Validation of the Nomogram

A nomogram integrating IPI components and the seven-gene model was established based on the GSE31312 cohort to assess the probability of 1-, 3-, and 5-year individualized OS *via* the rms R package (https://cran.r-project.org/web/packages/rms/). In addition, the discriminatory ability of the nomogram was graphically evaluated using a calibration map.

### CIBERSORT and Tumor-Infiltrating Immune Cell Profile

We used the CIBERSORT computational method to estimate the abundance distribution of TICs in GSE31312. The abundance of 22 immune cells was detected by t-test to observe the differences among risk groups. All analyses were performed with R (version 3.6.3, https://www.r-project.org/).

## Results

### Identification of Scores Associated With Survival and Clinical Features

**Figure 1:**
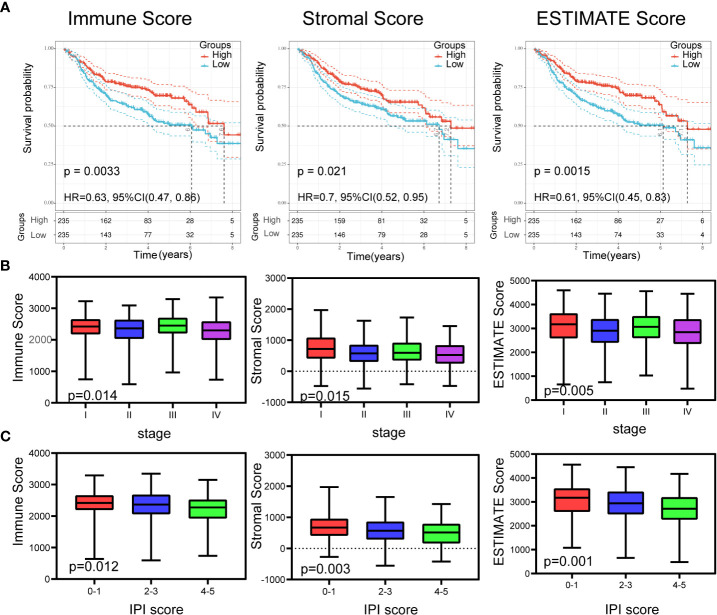
Relationship of scores with the survival and important clinical factor of patients with DLBCL. **(A)** Kaplan–Meier analysis for DLBCL patients grouped by ImmuneScores, StromalScores and ESTIMATEScore, and the differences between the two curves were determined by the logrank test. **(B)** The distribution of ImmuneScores, StromalScores and ESTIMATEScore in stages using Kruskal–Wallis rank sum test. **(C)** The distribution of ImmuneScores, StromalScores and ESTIMATEScore in IPI scores using Kruskal–Wallis rank sum test.

The clinical information of DLBCL patients from the GSE31312, GSE10846, and GSE11318 datasets is summarized in **Table 1**. To determine the correlations of ImmuneScore, StromalScore, and ESTIMATEScore with clinical features of DLBCL, clinical data from the GSE31312 dataset were analyzed. As shown in **Figure 1A**, the high-scoring group had longer OS than the low-scoring group for ImmuneScore, StromalScore, and ESTIMATEScore. Significant differences in ImmuneScore, StromalScore, and ESTIMATEScore were found at different stages of DLBCL by Kruskal–Wallis rank sum test (**Figure 1B**). On average, stage IV ranked the lowest among all stages with respect to ImmuneScore, StromalScore, and ESTIMATEScore (**Figure 1B**). Moreover, the scores showed a negative correlation with IPI scores by Kruskal–Wallis rank sum test (**Figure 1C**). Taken together, these results show that the immune and stromal components of the TME are related to prognosis and progression of DLBCL.

**Table 1:** The clinical characteristics of the DLBCL patients from GEO.

Characteristics	GSE31312(N = 470)	GSE10846(N = 414)	GSE11318(N = 200)
Age			
<=60	200 (42.55)	188 (45.41)	69 (34.5)
>60	270 (57.45)	226 (54.59)	94 (47)
NA			37 (18.5)
Gender			
Male	271 (57.66)	224 (54.11)	110 (55)
Female	199 (42.34)	172 (41.55)	90 (45)
NA		18 (4.34)	
Subtype			
GCB	227 (48.30)	183 (44.20)	70 (35)
ABC	199 (42.34)	167 (40.34)	73 (36.5)
NA	44 (9.36)	64 (15.46)	57 (28.5)
Stage			
I–II	220 (46.81)	189 (45.65)	75 (37.5)
IIII–IV	229 (48.72)	217 (52.42)	87 (43.5)
NA	21 (4.47)	8 (1.93)	38 (19)
ECOG			
<2	374 (79.57)	296 (71.50)	122 (61)
≥2	96 (20.43)	93 (22.46)	39 (19.5)
NA		25 (6.04)	39 (19.5)
LDH			
Normal	148 (31.50)	173 (41.78)	69 (34.5)
Elevated	278 (59.14)	178 (43.00)	78 (39)
NA	44 (9.36)	63 (15.22)	53 (26.5)
Extranodal sites			
<2	194 (41.28)	238 (57.49)	NA
≥2	276 (58.72)	145 (35.02)	
NA		31 (7.49)	
IPI score			
<2	169 (35.96)	NA	NA
≥2	255 (54.25)		
NA	46 (9.79)		

## DEGs and Enrichment Analysis

**Figure 2:**
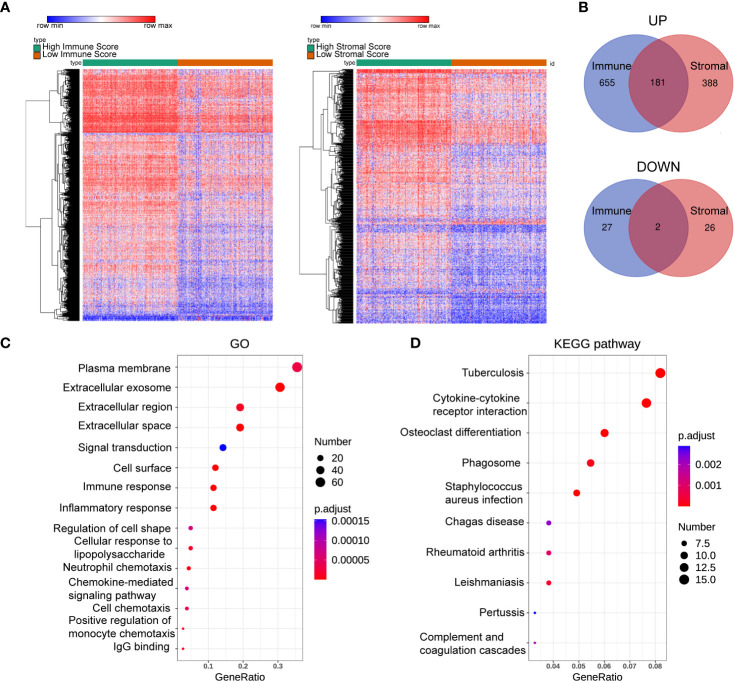
Heatmaps, Venn plots, and enrichment analysis. **(A)** The DEGs heatmaps are obtained by comparing the high score group and the low group in Immunescores and Stromalscores. **(B)** Venn diagram shows that there are 181 co-up-regulated gene and 2 co-down-regulated genes. **(C, D):** GO **(C)** and KEGG **(D)** analysis for 183 TME-related DEGs. number: Number of genes enriched. GO, Gene Ontology; KEGG, Kyoto Encyclopedia of Genes and Genomes.

To gain insight into the role of the TME in DLBCL, we divided patients into high-scoring and low-scoring groups according to the median ImmuneScore (StromalScore). The number of patients in the high ImmuneScore group who also had a high StromalScore group was 147, and the number of patients in the low ImmuneScore group who also had a low StromalScore group was also 147. Therefore, 294 patients had consistent ImmuneScore and StromalScore subgroups, representing 62.5% of all patients (**Supplementary Table S1**). We next investigated the changes in immune (or stromal) score-related DEGs between high-scoring and low-scoring samples. A total of 865 ImmuneScore-related DEGs were identified among which 836 genes were upregulated and 29 genes were downregulated (**Figure 2A**). Similarly, there were 597 StromalScore-related DEGs, including 569 upregulated and 28 downregulated genes (**Figure 2A**). A Venn diagram was used to depict the co-up/downregulated genes associated with the different score groups (N = 183, **Figure 2B**, **Supplementary Table S2**). Enrichment analyses showed that these DEGs were related to immune-related GO terms, including immune response and informatory response (**Figure 2C**, **Supplementary Table S3**). KEGG pathway analyses also indicated that the genes were mainly involved in cytokine-cytokine receptor interaction pathways (**Figure 2D**, **Supplementary Table S3**).

### Sharing of PPI Network and Univariate Cox Regression

**Figure 3:**
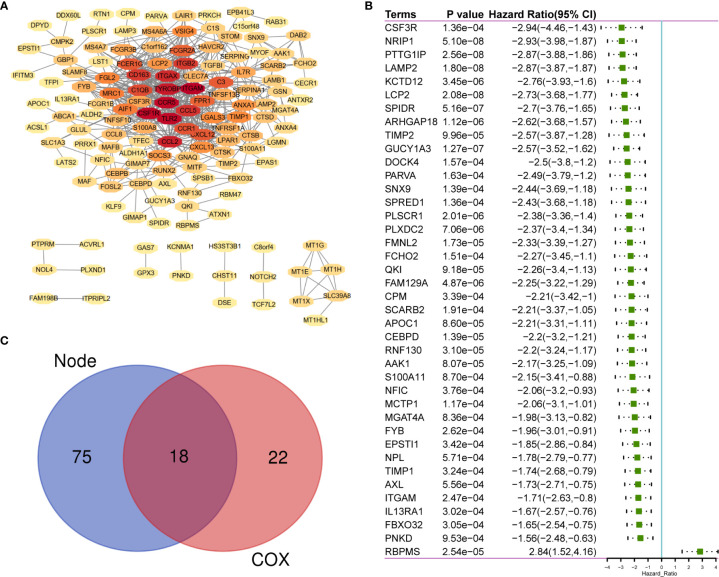
The sharing of PPI Network and Univariate COX Regression. **(A)** 183 TME-related DEGs were used to construct PPI network. **(B)** 183 DEGs was analyzed by univariate cox analysis, selecting the top significant factors with *p* < 0.0001. **(C)** 18 factors of overlap are obtained by venn map. PPI, Protein–protein interaction network.

A total of 183 DEGs were used to construct the PPI network, which is shown in **Figure 3A**. Univariate Cox regression was performed on 183 genes, and the genes with *p <*0.0001 were selected (**Figure 3B**). Then, intersection analysis was performed between the degree of the nodes ≥3 in the PPI and *p <*0.0001, resulting in 18 overlapping genes (**Figure 3C**).

### Construction of a Risk Prediction Model Based on the TME

**Figure 4:**
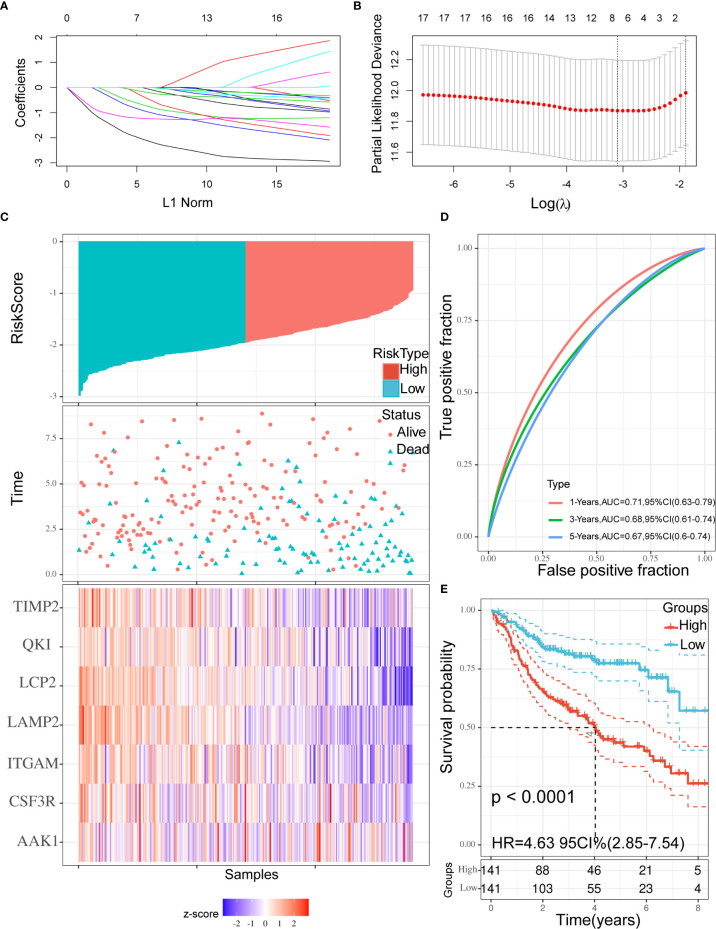
Construction of the prognostic signature. **(A, B)** Using 18 genes to perform a LASSO COX regression **(A)** and obtaining the seven-gene prognostic signature **(B)**. **(C)** The distribution of risk scores, the survival status of patients, and expression levels in training set were presented. **(D)** The time-dependent ROC curve and AUC of the signature. **(E)** Kaplan**–**Meier plots of overall survival between high- *vs* low-risk groups in training set by the logrank test. LASSO, Least absolute shrinkage and selection operator; ROC, Receiver operating characteristic curve; AUC, Area under curve.

According to the characteristics of variable selection and regularization, LASSO Cox regression was used to determine the optimal weight coefficient for the prognostic TME-related genes. Using one standard error of the best penalty parameter *λ* value and 1,000-fold cross-validation (**Figure 4A**), we obtained a seven-gene prognostic signature from the 18 genes identified above (**Figure 4B**). The left line indicated the optimal values by *λ*.min criteria (**Figure 4B**). Then, coefficient values were extracted, and the coefficients of the seven genes were multiplied by their mRNA expression levels to calculate individual risk scores using the following formula: Risk score = the mRNA expression level of CSF3R*(−0.06993781) + the mRNA expression level of QKI*(−0.76400334) + the mRNA expression level of LAMP2*(−2.14079252) + the mRNA expression level of TIMP2*(−0.98332426) + the mRNA expression level of LCP2*(−1.23980147) + the mRNA expression level of AAK1*(−0.46856491) + the mRNA expression level of ITGAM*(−0.20720812). Patients from the training group were divided into high-risk and low-risk groups based on the median risk score. The distributions of risk scores, survival status, and expression levels in the training set are presented in **Figure 4C**. Time-dependent ROC curve analysis showed that during 1-, 3-, and 5-year follow-up, the area under the curve (AUC) values were 0.71, 0.68, and 0.67 (**Figure 4D**). Survival analysis showed that patients in the high-risk group had significantly shorter median OS compared with the low-risk group (hazard ratio (HR) = 4.63; 95% confidence interval (CI) = 2.85–7.54; *p* < 0.0001; **Figure 4E**).

### Internal Validation of our Signature in GSE31312 Cohort

To verify the robustness of the seven-gene prognostic signature, we used the signature to calculate individual risk scores and divided patients from the GSE31312 cohort into high-risk and low-risk groups using the same cutoff value determined in the training set. The distributions of risk scores, survival status, and expression levels were consistent with those obtained in the training set (**Supplementary Figure S1A**). The verification results demonstrated that the AUC values were 0.71, 0.68, and 0.61 during 1-, 3-, and 5-year follow-up, respectively (**Supplementary Figure S1B**). The prognosis of the low-risk group was significantly better than that of the high-risk group (HR = 4.1; 95% CI = 2.3–7.33; *p* = 0.001; **Supplementary Figure S1C**). Finally, we also applied the seven-gene signature to all GSE31312 samples and found that the relationships between the distributions of risk scores, survival status, and expression levels were again consistent with those obtained in the training set (**Supplementary Figure S2A**). Time-dependent ROC curve analysis showed that in predicting 1-, 3-, and 5-year OS, the AUC values were 0.71, 0.68, and 0.65, respectively, indicating an acceptable degree of distinction (**Supplementary Figure S2B**). Similarly, the prognosis of the low-risk group was significantly better than that of the high-risk group (HR = 4.45; 95% CI = 3.07–6.47; *p* < 0.0001; **Supplementary Figure S2C**). Thus, the model effectively provided prognostic classifications within the GSE31312 dataset.

### External Validation of our Signature in GSE10846 and GSE11318 Cohorts

**Figure 5:**
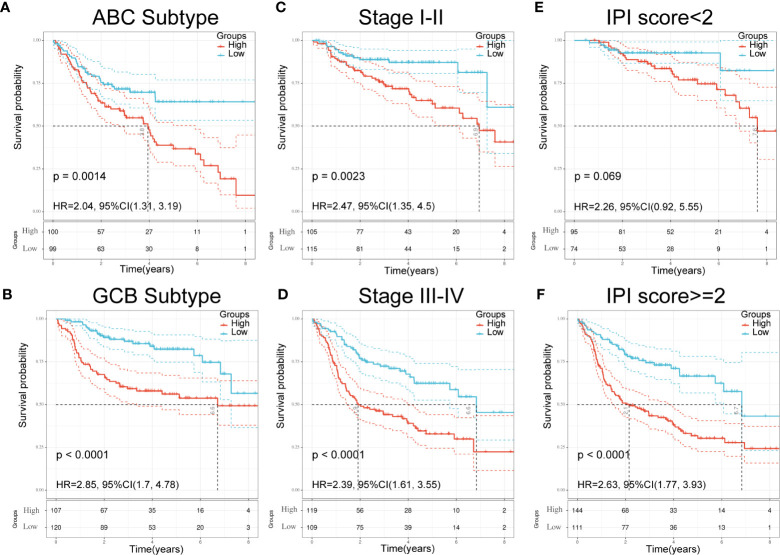
Validation of seven-gene signature in significant clinical subgroups. **(A, B)** Kaplan**–**Meier plots of overall survival between high and low risk groups in ABC subtype **(A)** and GCB subtype **(B)** by the logrank test. **(C, D):** Kaplan**–**Meier plots of overall survival between high- and low-risk groups in stages I**–**II **(C)** and III-IV **(D)** by the logrank test. **(E, F)** Kaplan**–**Meier plots of overall survival between high- and low-risk groups in IPI<2 **(E)** and IPI≥2 **(F)** by the logrank test.

The seven-gene prognostic signature was validated in the GSE10846 and GSE11318 datasets. The results are presented in **Figure 5** as Kaplan–Meier curves. Consistent with the above findings, there were significant differences in survival outcomes among different risk groups in both GSE10846 (HR = 1.96; 95% CI = 1.42–2.7; *p* < 0.0001; **Supplementary Figure S1D**) and GSE11318 (HR = 1.66; 95% CI = 1.14–2.42; *p* = 0.008; **Supplementary Figure S1E**). Thus, the signature was predictive in both internal and external datasets.

### Validation of the Seven-Gene Signature Using Clinical Parameters and Patient Outcomes

To further understand the relationship between the seven-gene signature and other clinical data, including pathologic subtype, clinical stage, and IPI score, we performed survival analysis using clinical parameters. The results demonstrated that low-risk patients had significantly favorable OS compared to high-risk patients with the activated B-cell-like (ABC) subtype of DLBCL (*p* = 0.0014; HR = 2.04; 95% CI = 1.31–3.19; **Figure 5A**). Similar results were obtained in germinal center B-cell-like (GCB) patients (*p* < 0.0001; HR = 2.85; 95% CI = 1.7–4.48; **Figure 5B**). An analogous result was also obtained in patients at different stages; the low-risk group had a favorable prognosis compared with the high-risk group (**Figures 5C, D** **)**. Moreover, patients in the low-risk group had significantly favorable OS compared with high-risk patients in both the IPI<2 group and the IPI≥2 group (**Figures 5E, F** **)**. These results demonstrate the independent predictive ability of our signature in clinical applications.

### Univariate and Multivariate Cox Regression for IPI Components and the Risk Prediction Model

To evaluate whether the risk prediction model could be used as an independent prognostic index for DLBCL patients, analyses were performed to identify the factors affecting the prognosis of DLBCL patients. These analyses were performed only on the datasets that included clinical IPI components data (GSE31312 and GSE10846), and the results showed that the seven-gene signature was an independent prognostic factor in these datasets (**Table 2**).

**Table 2:** Univariable and multivariable Cox regression analysis in DLBCL.

Variable	Univariate analysis			Multivariate analysis		
	HR	95% CI	P value	HR	95% CI	P value
**GSE31312**						
Our signature	2.603	1.878–3.608	0.000	2.954	2.058–4.24	0.000
Age (>60 vs. <=60 years)	1.849	1.336–2.56	0.000	1.871	1.319–2.655	0.000
ECOG (>=2 vs. <2)	2.036	1.46–2.84	0.000	1.617	1.107–2.363	0.013
Stage (III/IV vs. I/II)	2.337	1.687–3.237	0.000	2.08	1.406–3.077	0.000
LDH (Elevated vs. Normal)	2.129	1.452–3.121	0.000	1.496	1.003–2.231	0.048
Extranodal sites (>=2 vs. <2)	2.202	1.596–3.037	0.000	1.519	1.053–2.192	0.025
**GSE10846**						
Our signature	1.957	1.42–2.698	0.000	2.275	1.559–3.32	0.000
Age (>60 vs. <=60 years)	2.209	1.59–3.069	0.000	2.292	1.587–3.309	0.000
ECOG (>=2 vs. <2)	2.835	2.049–3.921	0.000	2.082	1.413–3.067	0.000
Stage (III/IV vs. I/II)	1.834	1.326–2.537	0.000	1.481	1.022–2.147	0.038
LDH (Elevated vs. Normal)	2.67	1.87–3.812	0.000	1.836	1.244–2.711	0.002
Extranodal sites (>=2 vs. <2)	1.927	1.144–3.246	0.014	1.095	0.575–2.085	0.782

CI, confidence interval; HR, hazard ratio.

### Construction and Validation of the Nomogram

**Figure 6:**
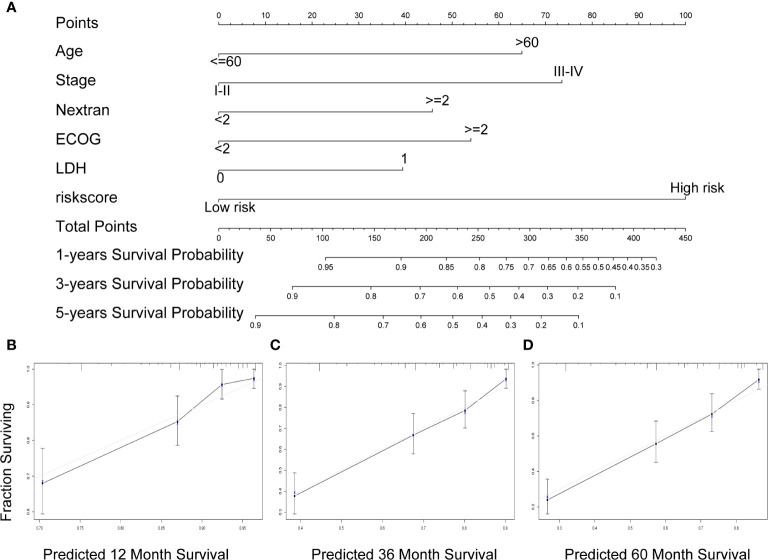
A nomogram Based on the seven-gene prognostic signature. **(A)** Construction of a nomogram to forecast 12-, 36-, and 60-month survival by the IPI components & the seven-gene model. **(B–D)** The calibration chart shows that the predicted survival rate is consistent with the actual survival rate for 12 months **(B)**, 36 months **(C)**, and 60 months **(D)**. IPI, International Prognostic Index.

A nomogram was established to forecast 1-, 3-, and 5-year survival based on IPI components and the seven-gene model (**Figure 6A**). The nomogram demonstrated that high total points predicted worse survival. The calibration chart showed an acceptable agreement between the predicted survival rate and the actual survival rate, indicating that our proposed nomogram has stability in predicting DLBCL patient prognosis in clinical practice (**Figures 6B–D** **)**.

### Immune-Infiltrating Cells and GSEA in Different Groups

**Figure 7:**
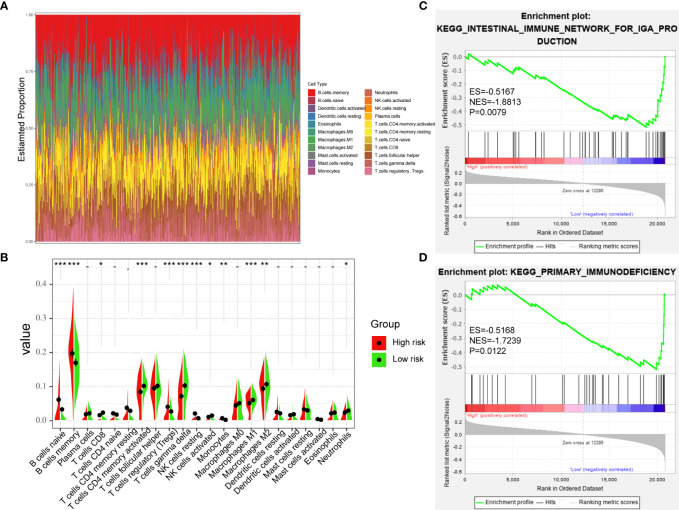
Immune-Infiltrating Cells and GSEA in Difference of Groups. **(A)** The landscape of immune cell infiltration of DLBCL patients in GSE31312. **(B)** the difference of 22 immune-infiltrating cells in GSE31312 samples among risk groups. **(C, D)** GSEA was performed between high-risk and low-risk groups based on the seven-gene signature. (*p < 0.05; **p < 0.01; ***p < 0.001).

In order to further verify the relationship between TME and DLBCL, we used CIBERSORT to analyze the proportions of 22 immune-infiltrating cells in GSE31312 samples between different risk groups. **Figure 7A** shows the compositions of immune cells in 470 patients, and **Figure 7B** shows the differences in the proportion of each immune cell type between the high-risk and low-risk groups. The results showed that 12 types of immune-infiltrating cells were correlated with the risk group. Specifically, the proportions of naïve B cells, memory B cells, regulatory T cells, resting natural killer (NK) cells, and monocytes were significantly higher in the high-risk group compared with the low-risk group, whereas the proportions of CD8+ T cells, activated CD4+ memory T cells, gamma delta T cells, activated NK cells, M1 macrophages, M2 macrophages, and neutrophils were significantly lower (**Figure 7B**). These results further confirmed that the prognostic signature was related to the immune activity of the TME. GSEA was used to probe the potential mechanism for the two risk groups to identify the enriched KEGG pathway. Two immune-related pathways were enriched in the low-risk group: intestinal immune network for IgA production and primary immunodeficiency diseases (**Figures 7C, D** **)**, and the rest of the enrichment results are shown in the **Supplementary Figure S1**. The immune pathway of the intestinal immune network for IgA production is associated with central deficits in the pathogenesis of the disease (14). Primary immunodeficiency is a pathway associated with primary immunodeficiency (15). These results suggest that the prognosis of DLBCL may be closely related to immune regulation in the TME.

## Discussion

DLBCL is characterized by a heterogeneous tumor entity with variable therapeutic outcomes. Risk stratification and prognosis of DLBCL patients remain challenges for clinicians and researchers (1, 16). In the rituximab era, IPI, R-IPI, and NCCN-IPI risk scores are calculated using easily obtained clinical features that are part of standard diagnostic procedures. However, all three scoring systems fail to identify a very high-risk group with long-term OS clearly below 50% (10). Moreover, the progression of a tumor is affected not only by its intrinsic characteristics but also by the extrinsic TME. Immune system accumulation and immune cell infiltration could have a profound impact on carcinogenesis and prognosis (17). There is also increasing evidence that the microenvironment has an important role in predicting tumor progression and prognosis (17, 18). Consequently, screening for a gene prognostic signature that adequately reflects the TME would be of great significance for individualized prevention and treatment of DLBCL patients (16, 19). In this study, we established seven prognostic gene markers which robustly and reliably predicted an individualized immune prognostic model for DLBCL patients on the basis of immune genes. The seven-gene prognostic signature was combined with IPI components to build a composite prognostic nomogram, which was capable of accurate prediction and showed positive net benefit in DLBCL.

The seven genes in our signature were *TIMP2*, *QKI*, *LCP2*, *LAMP2*, *ITGAM*, *CSF3R*, and *AAK1*. Although these genes are differentially expressed in immune cells or stromal cells, the expression of these genes in normal germinal center B cells and in a subset of DLBCL is uncertain, and our sequencing data comes from tumor tissues, so we cannot distinguish whether the expression level is caused by stromal or tumor components. High expression of *TIMP2* has been reported to inhibit matrix metalloproteinases to produce anti-tumor activity (20). *TIMP2* was also demonstrated to interact with multiple integrin pathways and is involved in angiogenesis in gastric cancer (21). *LCP2* encodes a protein of 533 amino acids that participates in T cell activation and increases the activity of the *IL-2* gene promoter through its transient overexpression (22). High expression of *LCP2* is associated with better outcomes in DLBCL patients (17, 18). *CSF3R* is closely related to prognosis of patients with chronic neutrophilic leukemia or atypical chronic myeloid leukemia and thus represents a potentially useful criterion for disease diagnosis (23). *QKI* gene encodes an RNA-binding protein that regulates the functions of diverse mRNAs, which play critical parts in tumorigenesis through inactivation of tumor suppressor genes in multiple tumors (24, 25). *LAMP2* is essential for maintaining the structural integrity of the lysosomal compartment and relocalizes to the cell surface of some highly metastatic tumor cells. *LAMP2* has been functionally validated as an essential mediator of drug resistance and tumor recurrence in hematological diseases (26–28). *ITGAM* and *AAK1* have not been previously reported to be related to cancer, and our study is the first to suggest that they could be used as new prognostic markers of DLBCL. Furthermore, the GSEA results showed that enrichment of the seven-gene signature was significantly correlated with immune-related signaling pathways, indicating that this model has potential clinical applications in predicting survival outcomes of patients.

Zamani-Ahmadmahmudi et al. constructed an independent seven-gene prognostic signature that could distinguish low-risk patients with DLBCL from high-risk ones (29). In our study, we not only constructed a risk prediction model for the prognosis of DLBCL patients, but we also explore the relationship between TME and DLBCL. The results showed that immune and stromal components in the TME were negatively correlated with the prognosis of DLBCL patients. Our TME-related seven-gene prognostic signature was shown to have strong predictive power and to represent an independent prognostic factor. In the era of immune targeting, distinguishing high-risk patients from the perspective of TME can inform clinical decisions and lead to better outcomes.

Although our TME-based prognostic model was shown to predict tumor prognosis in a large sample, this study had some limitations. First, owing to the retrospective design and the unavailability of control group samples in the GEO databases, the results were biased to some extent. Thus, a well-designed, prospective, international, multicenter clinical trial is needed to verify our findings in the future. In addition, further functional research is warranted to explore the molecular functions of the identified immune genes during DLBCL progression.

In conclusion, we established for the first time a TME-related prognostic signature in DLBCL patients, which is a promising prognostic model when combined with clinical IPI components. The results presented here not only help to clarify immune responses in the DLBCL microenvironment but also indicate new clinical applications for immune therapy and individualized therapy in patients with DLBCL.

## Data Availability Statement

Publicly available data sets were analyzed in this study. These data can be found here: https://www.ncbi.nlm.nih.gov/geo/.

## Author Contributions

HZ and LX conceived and designed the study and reviewed the manuscript. TP and YH collected, arranged, and analyzed the data and wrote the manuscript. HC, YL, RZ, and JX revised the statistical methodology. JP, SC, YZ, and LQ designed and prepared the figures and tables. All authors contributed to the article and approved the submitted version.

## Funding

This study was supported by grants from the National Natural Science Foundation of China [no. 82000200], Natural Science Foundation of Hunan Provincial Health Commission [no. 20201659], The Research Program of Hunan provincial health and family planning commission [no: B20180496], “Scientific Research Climbing Plan” of Hunan Cancer Hospital [No: ZX2020003], The Changsha Science and Technology plan [no: kq1706041], and Fundamental Research Funds for the Central Universities of Central South University [nos 2019zzts1002 and 2019zzts1060].

## Conflict of Interest

The authors declare that the research was conducted in the absence of any commercial or financial relationships that could be construed as a potential conflict of interest.

## Supplementary Material

Supplementary Figure 1 Validation of the prognostic signature. **(A)** The distribution of risk scores, the survival status of patients, and expression levels in internal validation set were presented. **(B)** The time-dependent ROC curve and AUC in internal validation set. **(C)** Kaplan-Meier plots of overall survival between high- *vs* low-risk internal validation groups by the logrank test. **(D)** Kaplan–Meier plots of overall survival between high- and low-risk groups in GSE10846 by the logrank test. **(E)** Kaplan–Meier plots of overall survival between high- and low-risk groups in GSE11318 by the logrank test. ROC, receiver operating characteristic curve; AUC, area under curve.

Supplementary Figure 2 Validation of the prognostic signature in all GSE31312 tumor samples. **(A)** The distribution of risk scores, the survival status of patients, and expression levels were presented. **(B)** The time-dependent ROC curve and AUC in all GSE31312 patients. **(C)** Kaplan–Meier plots of overall survival between high- and low-risk groups in GSE31312 by the logrank test. ROC, receiver operating characteristic curve; AUC, area under curve.

Supplementary Table 1 ImmuneScores, StromalScores, and ESTIMATEScore.

Supplementary Table 2 The list of 183 DEGs.

Supplementary Table 3 The results of the gene enrichment analysis.

Supplementary Picture 1 GSEA enrichment results.
