# Supplementary figures and images for: Identification and Validation of a Prognostic Gene Signature for Diffuse Large B-Cell Lymphoma Based on Tumor Microenvironment-Related Genes
Source: Front Oncol. 2021 Feb 22;11:614211. doi: 10.3389/fonc.2021.614211 (PMC7938316; doi:10.3389/fonc.2021.614211)

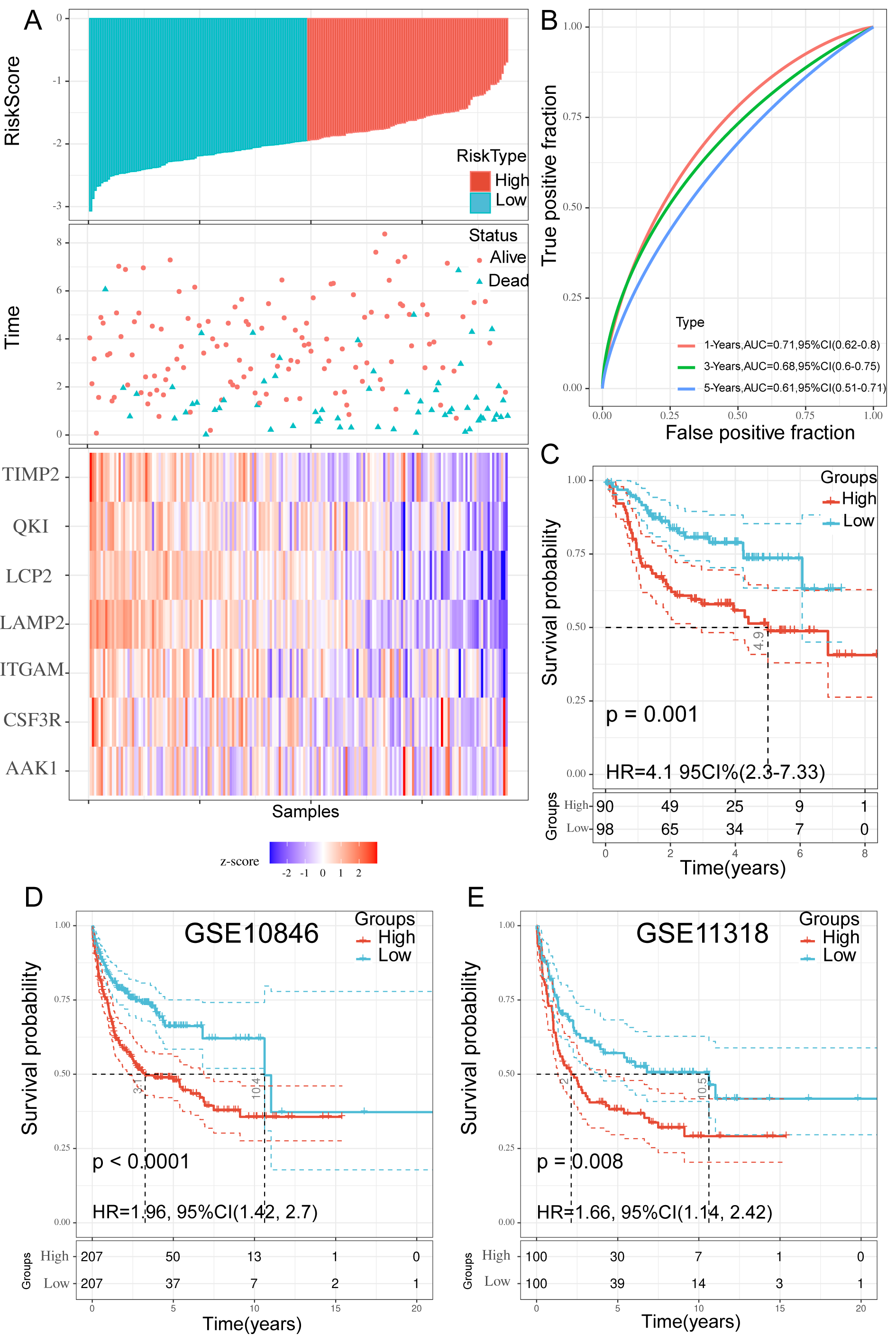

Supplement: Supplementary Figure 1 — Validation of the prognostic signature. (A) The distribution of risk scores, the survival status of patients, and expression levels in internal validation set were presented. (B) The time-dependent ROC curve and AUC in internal validation set. (C) Kaplan-Meier plots of overall survival between high- vs low-risk internal validation groups by the logrank test. (D) Kaplan–Meier plots of overall survival between high- and low-risk groups in GSE10846 by the logrank test. (E) Kaplan–Meier plots of overall survival between high- and low-risk groups in GSE11318 by the logrank test. ROC, receiver operating characteristic curve; AUC, area under curve. [file Image_1.tif]

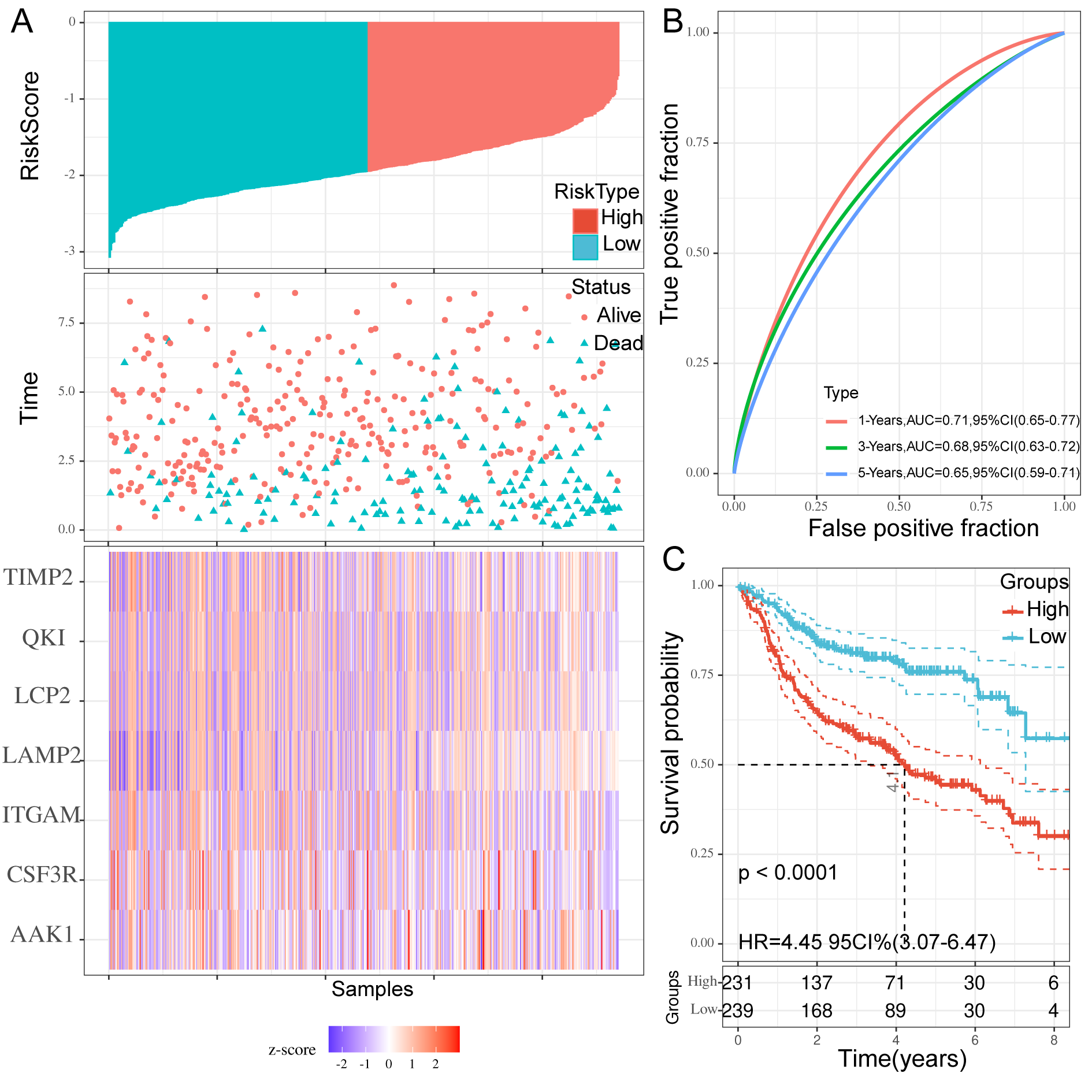

Supplement: Supplementary Figure 2 — Validation of the prognostic signature in all GSE31312 tumor samples. (A) The distribution of risk scores, the survival status of patients, and expression levels were presented. (B) The time-dependent ROC curve and AUC in all GSE31312 patients. (C) Kaplan–Meier plots of overall survival between high- and low-risk groups in GSE31312 by the logrank test. ROC, receiver operating characteristic curve; AUC, area under curve. [file Image_2.tif]

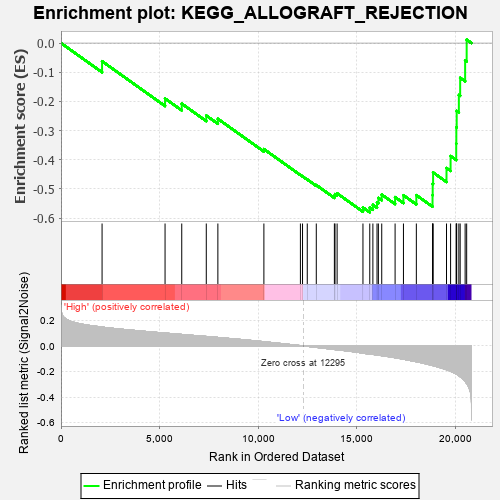

Supplement: Supplementary Picture 1 — GSEA enrichment results. [file DataSheet_1.zip › enplot_KEGG_ALLOGRAFT_REJECTION_164.png]

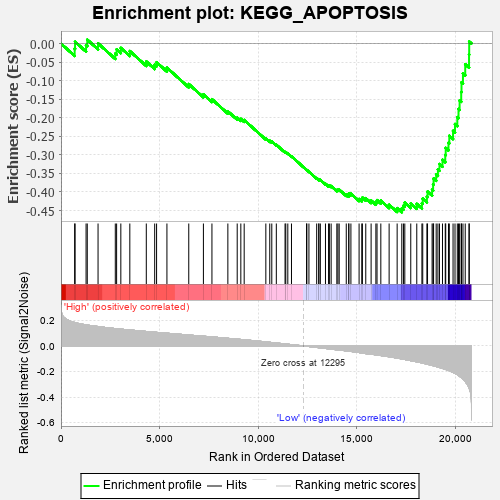

Supplement: Supplementary Picture 1 — GSEA enrichment results. [file DataSheet_1.zip › enplot_KEGG_APOPTOSIS_173.png]

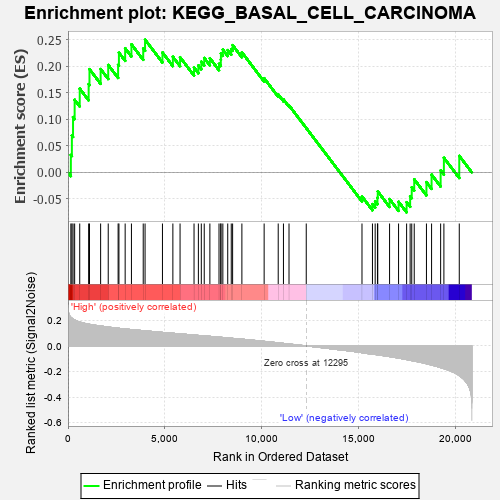

Supplement: Supplementary Picture 1 — GSEA enrichment results. [file DataSheet_1.zip › enplot_KEGG_BASAL_CELL_CARCINOMA_125.png]

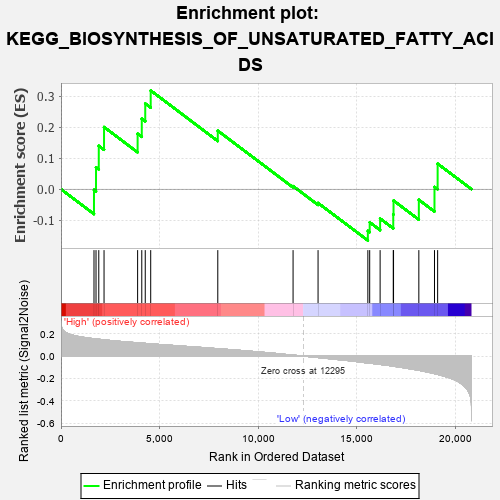

Supplement: Supplementary Picture 1 — GSEA enrichment results. [file DataSheet_1.zip › enplot_KEGG_BIOSYNTHESIS_OF_UNSATURATED_FATTY_ACIDS_116.png]

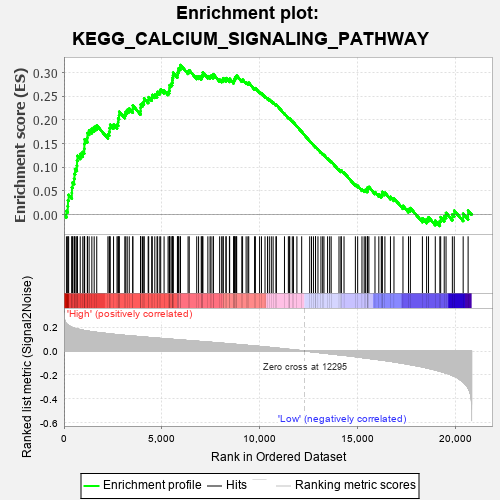

Supplement: Supplementary Picture 1 — GSEA enrichment results. [file DataSheet_1.zip › enplot_KEGG_CALCIUM_SIGNALING_PATHWAY_89.png]

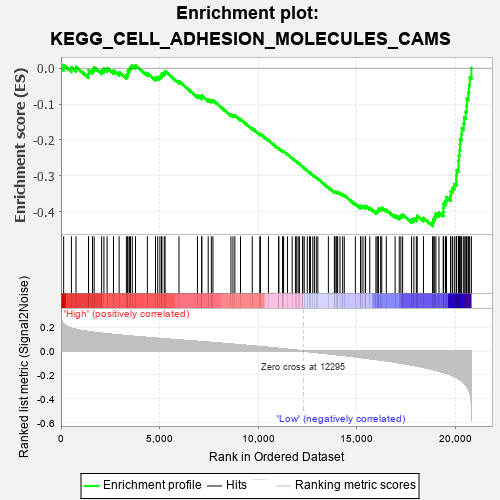

Supplement: Supplementary Picture 1 — GSEA enrichment results. [file DataSheet_1.zip › enplot_KEGG_CELL_ADHESION_MOLECULES_CAMS_188.png]

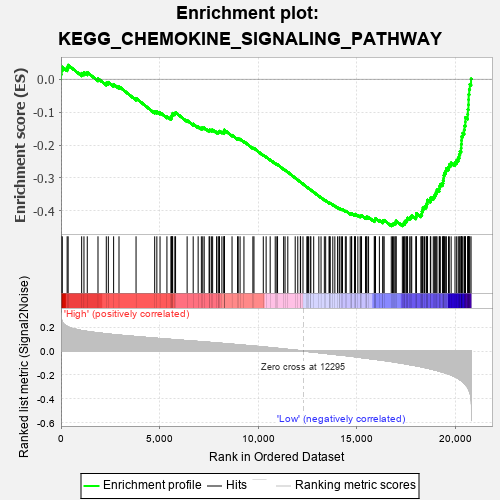

Supplement: Supplementary Picture 1 — GSEA enrichment results. [file DataSheet_1.zip › enplot_KEGG_CHEMOKINE_SIGNALING_PATHWAY_158.png]

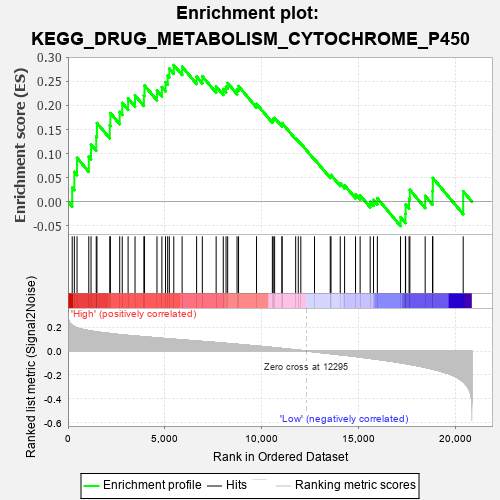

Supplement: Supplementary Picture 1 — GSEA enrichment results. [file DataSheet_1.zip › enplot_KEGG_DRUG_METABOLISM_CYTOCHROME_P450_110.png]

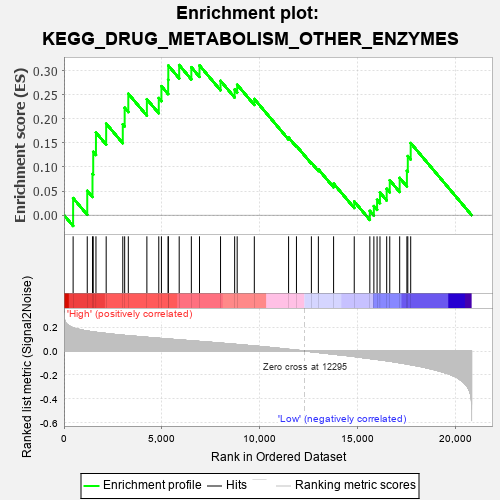

Supplement: Supplementary Picture 1 — GSEA enrichment results. [file DataSheet_1.zip › enplot_KEGG_DRUG_METABOLISM_OTHER_ENZYMES_107.png]

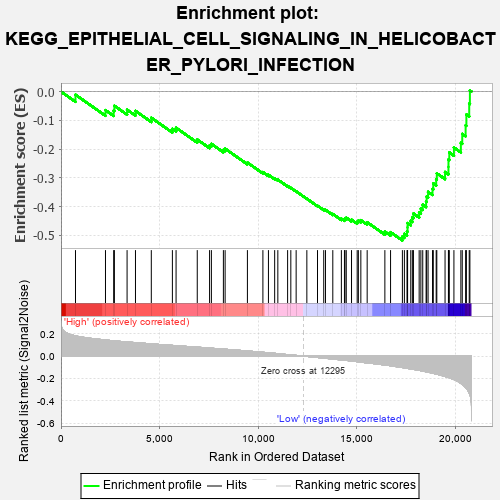

Supplement: Supplementary Picture 1 — GSEA enrichment results. [file DataSheet_1.zip › enplot_KEGG_EPITHELIAL_CELL_SIGNALING_IN_HELICOBACTER_PYLORI_INFECTION_149.png]

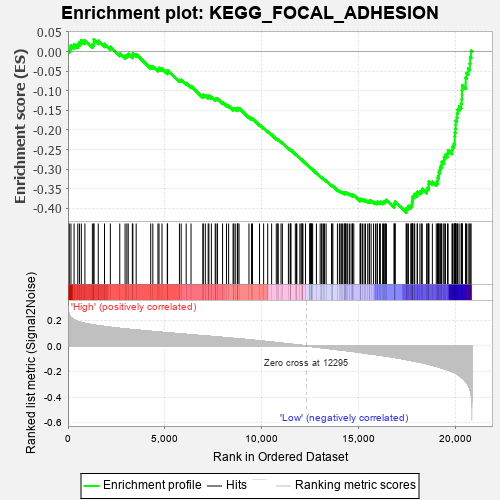

Supplement: Supplementary Picture 1 — GSEA enrichment results. [file DataSheet_1.zip › enplot_KEGG_FOCAL_ADHESION_182.png]

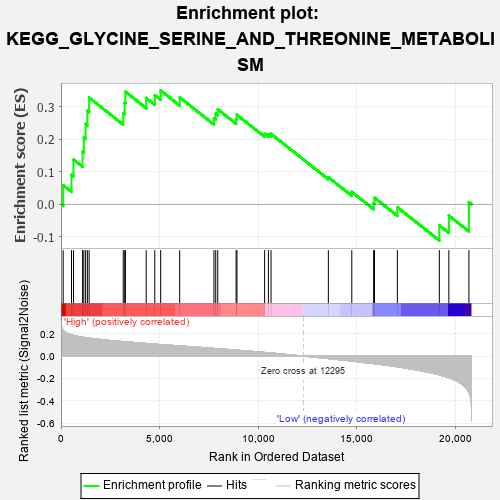

Supplement: Supplementary Picture 1 — GSEA enrichment results. [file DataSheet_1.zip › enplot_KEGG_GLYCINE_SERINE_AND_THREONINE_METABOLISM_98.png]

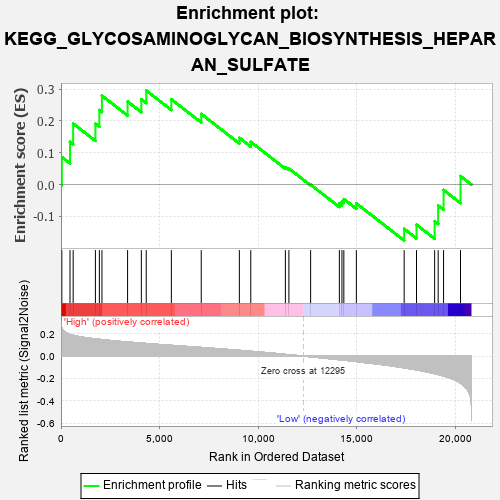

Supplement: Supplementary Picture 1 — GSEA enrichment results. [file DataSheet_1.zip › enplot_KEGG_GLYCOSAMINOGLYCAN_BIOSYNTHESIS_HEPARAN_SULFATE_113.png]

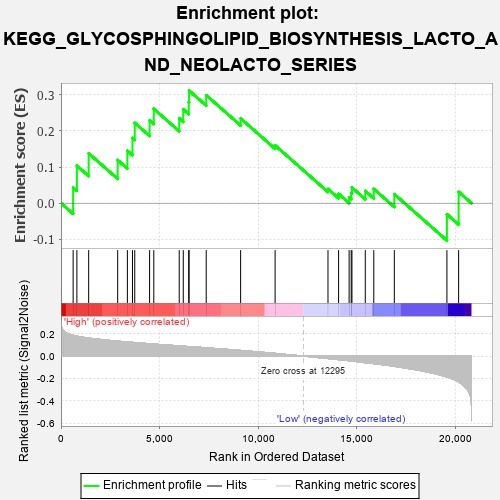

Supplement: Supplementary Picture 1 — GSEA enrichment results. [file DataSheet_1.zip › enplot_KEGG_GLYCOSPHINGOLIPID_BIOSYNTHESIS_LACTO_AND_NEOLACTO_SERIES_119.png]

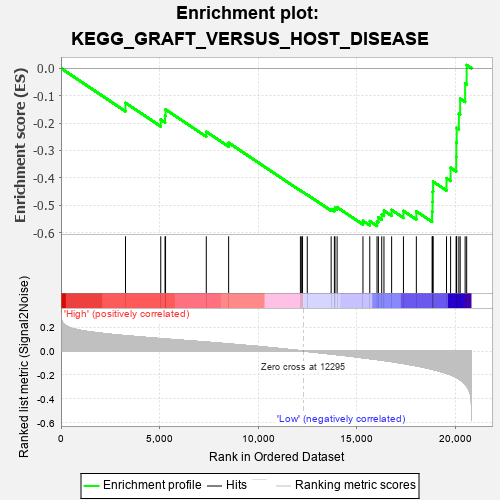

Supplement: Supplementary Picture 1 — GSEA enrichment results. [file DataSheet_1.zip › enplot_KEGG_GRAFT_VERSUS_HOST_DISEASE_170.png]

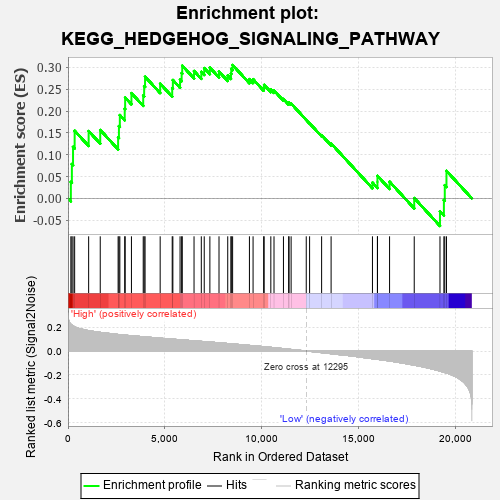

Supplement: Supplementary Picture 1 — GSEA enrichment results. [file DataSheet_1.zip › enplot_KEGG_HEDGEHOG_SIGNALING_PATHWAY_104.png]

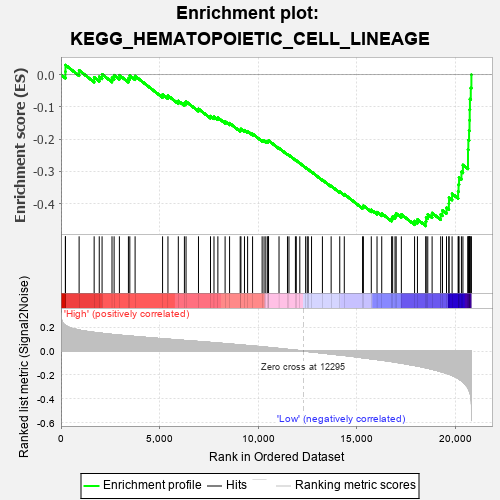

Supplement: Supplementary Picture 1 — GSEA enrichment results. [file DataSheet_1.zip › enplot_KEGG_HEMATOPOIETIC_CELL_LINEAGE_176.png]

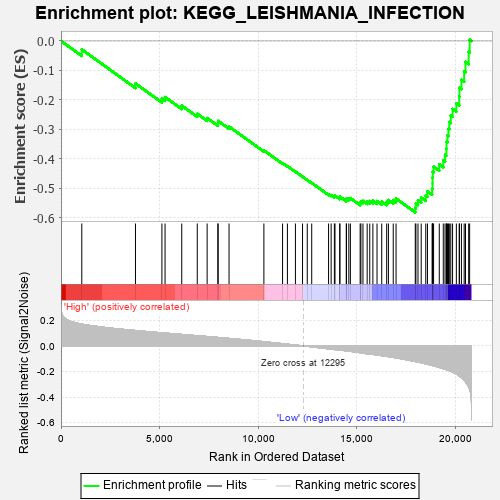

Supplement: Supplementary Picture 1 — GSEA enrichment results. [file DataSheet_1.zip › enplot_KEGG_LEISHMANIA_INFECTION_143.png]

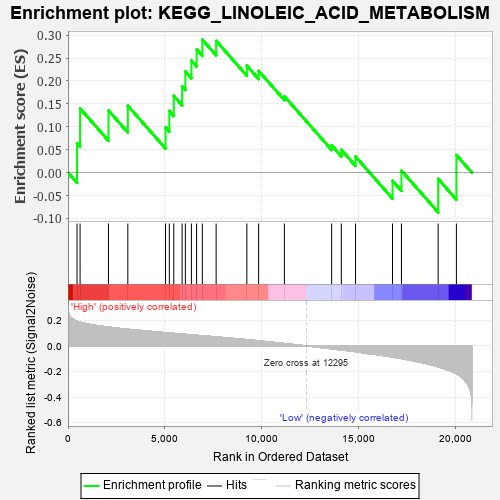

Supplement: Supplementary Picture 1 — GSEA enrichment results. [file DataSheet_1.zip › enplot_KEGG_LINOLEIC_ACID_METABOLISM_128.png]

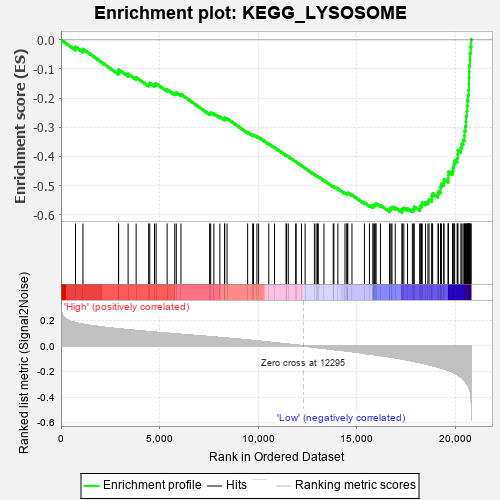

Supplement: Supplementary Picture 1 — GSEA enrichment results. [file DataSheet_1.zip › enplot_KEGG_LYSOSOME_137.png]

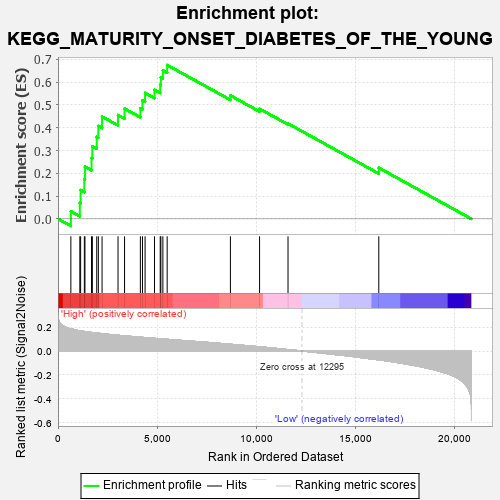

Supplement: Supplementary Picture 1 — GSEA enrichment results. [file DataSheet_1.zip › enplot_KEGG_MATURITY_ONSET_DIABETES_OF_THE_YOUNG_77.png]

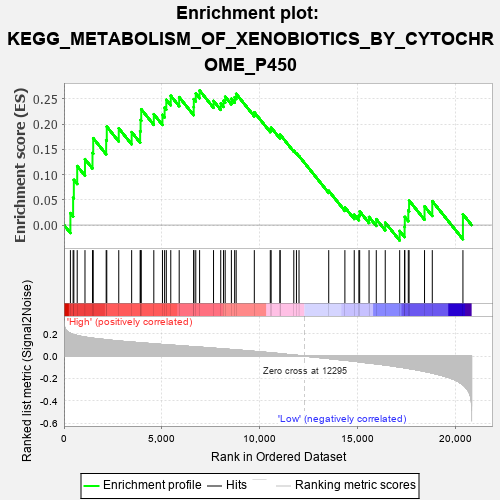

Supplement: Supplementary Picture 1 — GSEA enrichment results. [file DataSheet_1.zip › enplot_KEGG_METABOLISM_OF_XENOBIOTICS_BY_CYTOCHROME_P450_122.png]

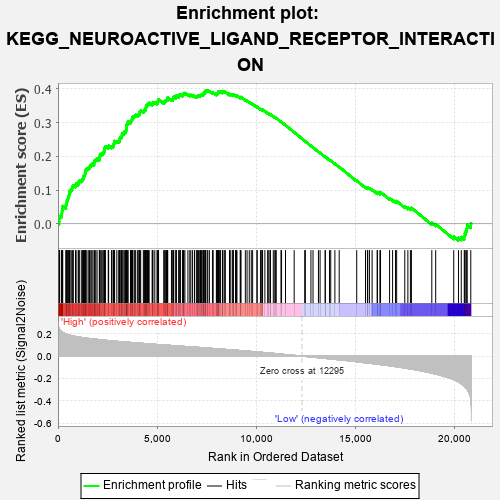

Supplement: Supplementary Picture 1 — GSEA enrichment results. [file DataSheet_1.zip › enplot_KEGG_NEUROACTIVE_LIGAND_RECEPTOR_INTERACTION_83.png]

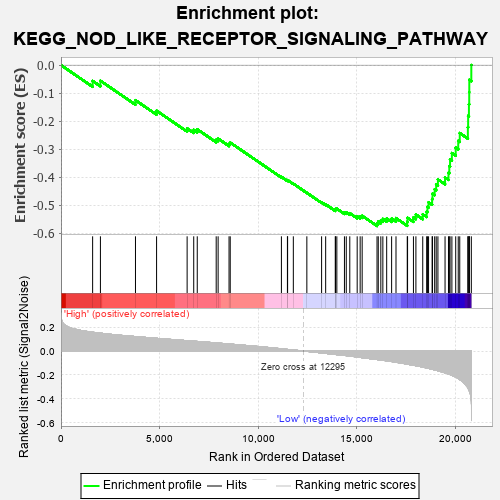

Supplement: Supplementary Picture 1 — GSEA enrichment results. [file DataSheet_1.zip › enplot_KEGG_NOD_LIKE_RECEPTOR_SIGNALING_PATHWAY_146.png]

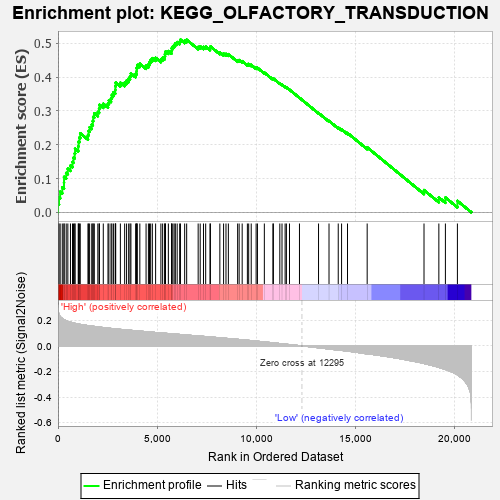

Supplement: Supplementary Picture 1 — GSEA enrichment results. [file DataSheet_1.zip › enplot_KEGG_OLFACTORY_TRANSDUCTION_80.png]

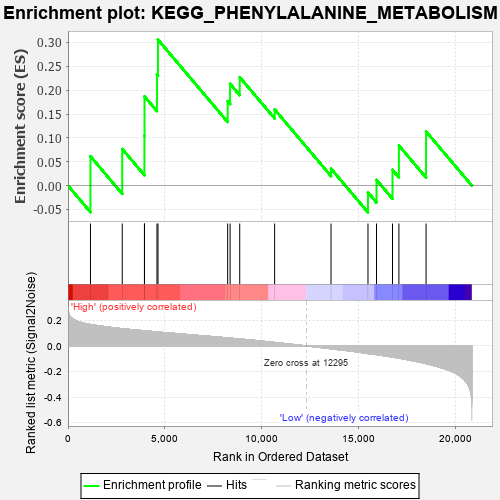

Supplement: Supplementary Picture 1 — GSEA enrichment results. [file DataSheet_1.zip › enplot_KEGG_PHENYLALANINE_METABOLISM_134.png]

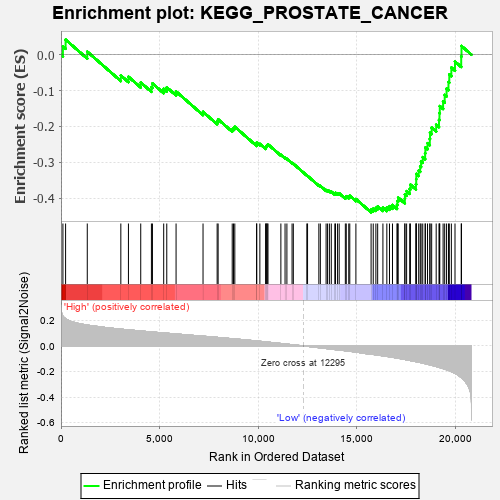

Supplement: Supplementary Picture 1 — GSEA enrichment results. [file DataSheet_1.zip › enplot_KEGG_PROSTATE_CANCER_179.png]

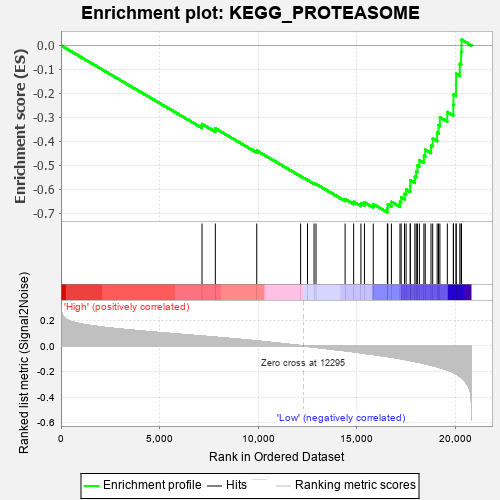

Supplement: Supplementary Picture 1 — GSEA enrichment results. [file DataSheet_1.zip › enplot_KEGG_PROTEASOME_152.png]

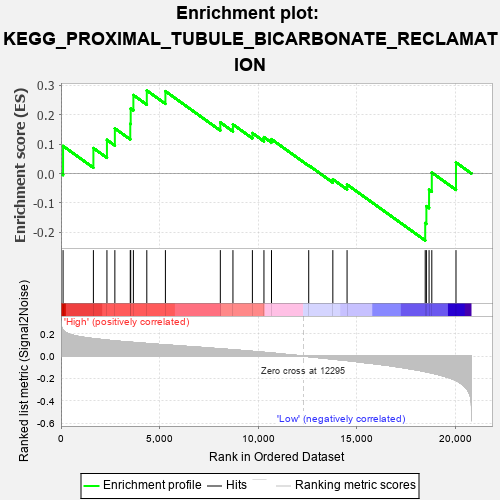

Supplement: Supplementary Picture 1 — GSEA enrichment results. [file DataSheet_1.zip › enplot_KEGG_PROXIMAL_TUBULE_BICARBONATE_RECLAMATION_131.png]

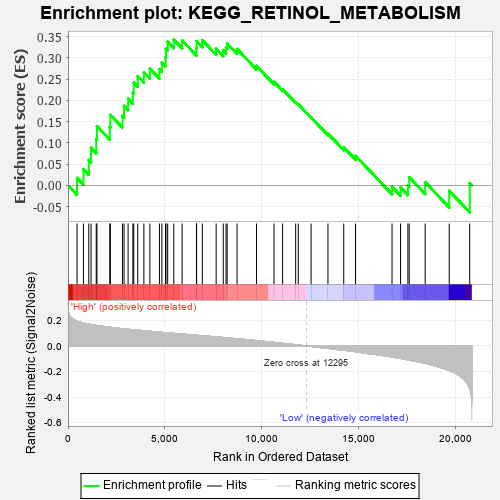

Supplement: Supplementary Picture 1 — GSEA enrichment results. [file DataSheet_1.zip › enplot_KEGG_RETINOL_METABOLISM_101.png]

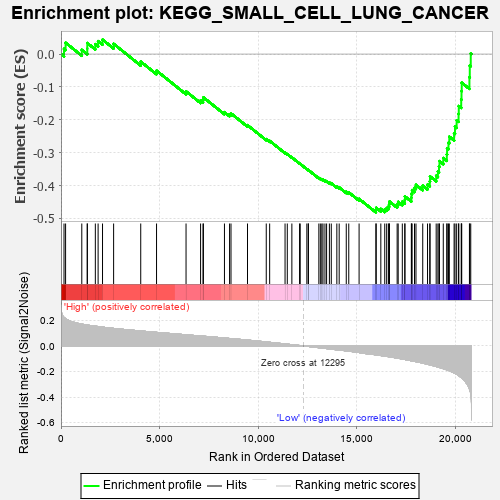

Supplement: Supplementary Picture 1 — GSEA enrichment results. [file DataSheet_1.zip › enplot_KEGG_SMALL_CELL_LUNG_CANCER_161.png]

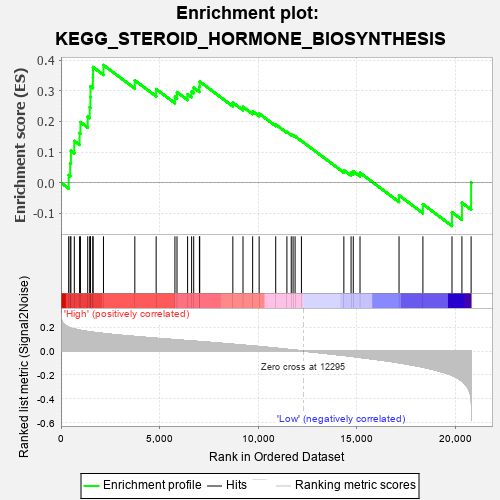

Supplement: Supplementary Picture 1 — GSEA enrichment results. [file DataSheet_1.zip › enplot_KEGG_STEROID_HORMONE_BIOSYNTHESIS_95.png]

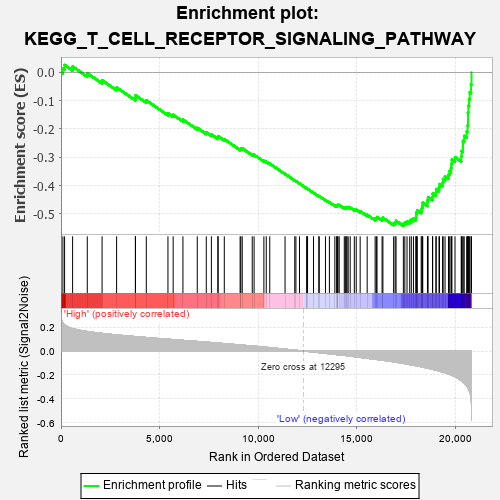

Supplement: Supplementary Picture 1 — GSEA enrichment results. [file DataSheet_1.zip › enplot_KEGG_T_CELL_RECEPTOR_SIGNALING_PATHWAY_140.png]

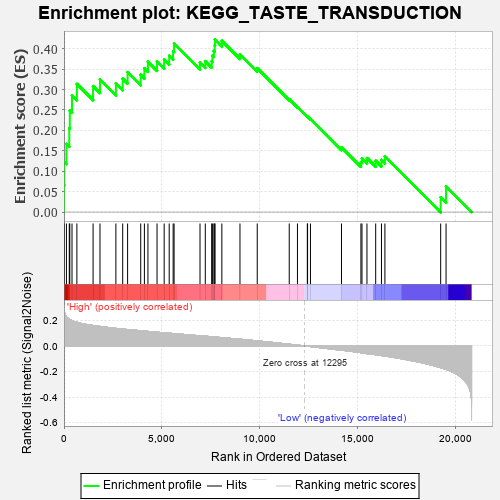

Supplement: Supplementary Picture 1 — GSEA enrichment results. [file DataSheet_1.zip › enplot_KEGG_TASTE_TRANSDUCTION_86.png]

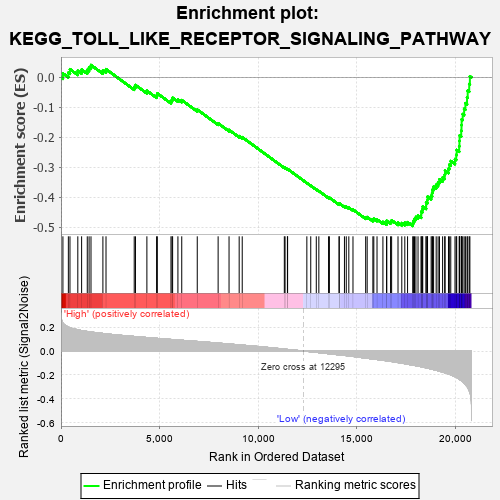

Supplement: Supplementary Picture 1 — GSEA enrichment results. [file DataSheet_1.zip › enplot_KEGG_TOLL_LIKE_RECEPTOR_SIGNALING_PATHWAY_155.png]

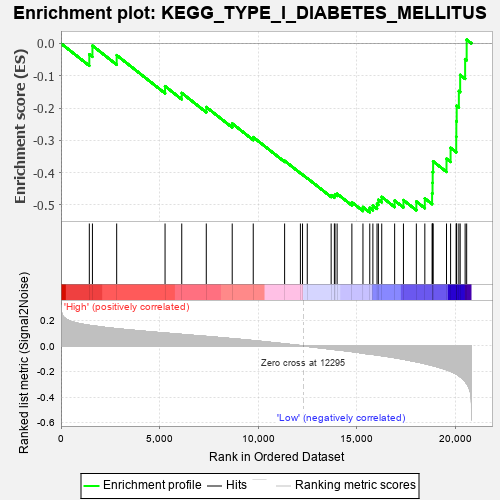

Supplement: Supplementary Picture 1 — GSEA enrichment results. [file DataSheet_1.zip › enplot_KEGG_TYPE_I_DIABETES_MELLITUS_185.png]

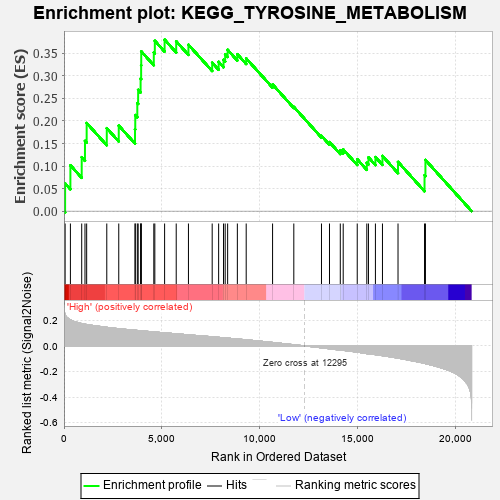

Supplement: Supplementary Picture 1 — GSEA enrichment results. [file DataSheet_1.zip › enplot_KEGG_TYROSINE_METABOLISM_92.png]

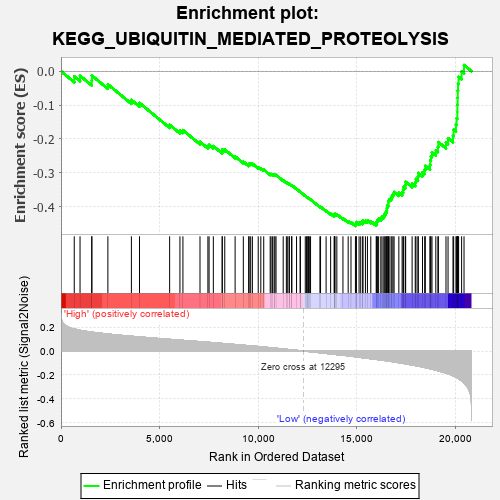

Supplement: Supplementary Picture 1 — GSEA enrichment results. [file DataSheet_1.zip › enplot_KEGG_UBIQUITIN_MEDIATED_PROTEOLYSIS_191.png]
