# Supplementary material for: Identification and Validation of a Prognostic Gene Signature for Diffuse Large B-Cell Lymphoma Based on Tumor Microenvironment-Related Genes
Source: Front Oncol. 2021 Feb 22;11:614211. doi: 10.3389/fonc.2021.614211 (PMC7938316; doi:10.3389/fonc.2021.614211)
Supplement: Supplementary Table 3 — The results of the gene enrichment analysis. [file Table_3.docx]

| **Category** | **Term** | **Genes** | **P Value** |
| --- | --- | --- | --- |
| Biological process | GO:0006954~inflammatory response | HAVCR2, CCL2, ADGRE2, CEBPB, S100A8, C3, AIF1, CCR1, ANXA1, TLR2, FPR1, AXL, CCL8, ITGB2, CCL5, CXCL12, TNFRSF1A, CCR5, CXCL13, CLEC7A, CSF1R | 8.95E-10 |
| Biological process | GO:0006955~immune response | FYB, CCL2, LST1, CEBPB, C3, IFITM3, CCR1, TLR2, CCL8, IL7R, CCL5, CXCL12, TNFRSF1A, TNFSF10, TNFSF13B, CCR5, CXCL13, FCGR2C, FCGR1B, FCGR3B, LCP2 | 5.44E-09 |
| Cellular component | GO:0009986~cell surface | HAVCR2, MRC1, PAM, CPM, ACVRL1, ANXA1, AXL, TLR2, ITGB2, LPAR1, TIMP2, ANXA4, ITGAM, NOTCH2, TNFRSF1A, SLC1A3, ITGAX, CCR5, TFPI, FCER1G, TYROBP, CSF1R | 7.30E-08 |
| Cellular component | GO:0070062~extracellular exosome | ARSD, S100A8, LGMN, CXCL12, DAB2, GSN, TGFBI, GPX3, SERPINA1, FAM129A, FCGR3B, KCNMA1, LAIR1, PRKCH, SERPING1, STOM, C1QB, GLUL, GNAQ, PLXDC2, CTSD, CTSB, VSIG4, SNX9, PAM, CPM, C3, IFITM3, APOC1, ITGB2, C1S, TIMP2, ITGAM, TIMP1, ALDH1A1, GLIPR2, HNMT, FGL2, SCARB2, LAMB1, MYOF, HAVCR2, MGAT4A, LGALS3, S100A11, AXL, ANXA1, ANXA4, MXRA5, PLSCR1, LAMP2, TNFSF10, ALDH2, PTTG1IP, FCGR2A, KCTD12 | 1.17E-07 |
| Cellular component | GO:0005615~extracellular space | PAM, CPM, CCL2, S100A8, C3, CCL8, TIMP2, CCL5, CXCL12, ABI3BP, ITGAM, TIMP1, TNFRSF1A, DMXL2, GSN, TGFBI, GPX3, SERPINA1, LAMB1, LGALS3, ANXA1, S100A11, CECR1, AXL, SERPING1, STOM, DKK3, CTSK, LAMP2, TNFSF10, TNFSF13B, CXCL13, TFPI, CTSD, CTSB | 2.23E-07 |
| Molecular function | GO:0019864~IgG binding | FCGR2C, FCGR1B, FCER1G, FCGR2A, FCGR3B | 2.89E-06 |
| Biological process | GO:0030593~neutrophil chemotaxis | CCL2, S100A8, LGALS3, CCL8, FCER1G, CSF3R, ITGB2, CCL5 | 3.58E-06 |
| Cellular component | GO:0005576~extracellular region | CCL2, S100A8, C3, APOC1, C1S, TIMP2, CCL5, IL7R, CXCL12, TIMP1, GLIPR2, TNFRSF1A, GSN, GPX3, TGFBI, CSF3R, SERPINA1, LAMB1, ANXA1, CECR1, SERPING1, CD163, NOTCH2, DKK3, C1QB, CTSK, TNFSF10, TNFSF13B, CXCL13, TFPI, CTSD, ANTXR2, CTSB, ADAMTS2, GBP1 | 1.20E-05 |
| Biological process | GO:0090026~positive regulation of monocyte chemotaxis | CCL2, AIF1, CCR1, CCL5, CXCL12 | 1.46E-05 |
| Biological process | GO:0071222~cellular response to lipopolysaccharide | HAVCR2, MRC1, CCL2, CEBPB, CCR5, TFPI, AXL, ABCA1, CMPK2 | 1.50E-05 |
| Cellular component | GO:0005886~plasma membrane | ACVRL1, S100A8, TLR2, LPAR1, DAB2, SLC1A3, GSN, TGFBI, CSF3R, GUCY1A3, SPRED1, FAM129A, IL13RA1, FCGR3B, KCNMA1, LAIR1, ADGRE2, PRKCH, CD163, EPB41L3, TNFSF13B, CCR5, GNAQ, TFPI, GBP1, PARVA, SNX9, PAM, CPM, C3, IFITM3, CCR1, FPR1, ITGB2, ABCA1, IL7R, ITGAM, TNFRSF1A, ACSL1, ITGAX, FCGR1B, SLC39A8, FCER1G, PLXND1, FCHO2, MYOF, TYROBP, CSF1R, MRC1, FYB, LGALS3, KLF9, AXL, ANXA1, ANXA4, DOCK4, FNIP2, NOTCH2, PLSCR1, LAMP2, LAMP3, FCGR2C, ANTXR2, CLEC7A, FCGR2A | 2.81E-05 |
| Biological process | GO:0060326~cell chemotaxis | CCL2, FPR1, CCL8, LPAR1, CCL5, CXCL12, DOCK4 | 4.06E-05 |
| Biological process | GO:0070098~chemokine-mediated signaling pathway | CCL2, CCR5, CXCL13, CCR1, CCL8, CCL5, CXCL12 | 6.71E-05 |
| Biological process | GO:0008360~regulation of cell shape | EPB41L3, LST1, CCL2, ANXA1, ARHGAP18, ITGB2, LPAR1, CSF1R, PARVA | 7.00E-05 |
| Biological process | GO:0007165~signal transduction | ACVRL1, CCL2, C3, TLR2, FPR1, CCL8, IL7R, CXCL12, TNFRSF1A, CSF3R, CSF1R, TYROBP, FYB, MRC1, PTPRM, EPAS1, ANXA1, AXL, S100A11, PRKCH, ANXA4, RASSF4, TNFSF10, TNFSF13B, FCGR2C, RIN2 | 1.54E-04 |
| KEGG_PATHWAY | hsa05152:Tuberculosis | MRC1, CEBPB, C3, TLR2, ITGB2, ITGAM, TNFRSF1A, LAMP2, ITGAX, FCGR2C, FCER1G, CTSD, FCGR2A, CLEC7A, FCGR3B | 5.48E-08 |
| KEGG_PATHWAY | hsa05150:Staphylococcus aureus infection | C1QB, C3, FCGR2C, FPR1, ITGB2, FCGR2A, C1S, FCGR3B, ITGAM | 3.99E-07 |
| KEGG_PATHWAY | hsa04380:Osteoclast differentiation | TNFRSF1A, CTSK, FOSL2, SOCS3, FCGR2C, MITF, FCGR2A, FCGR3B, CSF1R, TYROBP, LCP2 | 6.99E-06 |
| KEGG_PATHWAY | hsa04060:Cytokine-cytokine receptor interaction | CCL2, CCR1, CCL8, IL7R, CCL5, CXCL12, TNFRSF1A, TNFSF10, CCR5, TNFSF13B, CXCL13, CSF3R, IL13RA1, CSF1R | 1.41E-05 |
| KEGG_PATHWAY | hsa04145:Phagosome | MRC1, LAMP2, C3, FCGR2C, TLR2, ITGB2, FCGR2A, CLEC7A, FCGR3B, ITGAM | 1.37E-04 |
| KEGG_PATHWAY | hsa05140:Leishmaniasis | C3, FCGR2C, TLR2, ITGB2, FCGR2A, FCGR3B, ITGAM | 3.07E-04 |
| KEGG_PATHWAY | hsa05323:Rheumatoid arthritis | CTSK, CCL2, TNFSF13B, TLR2, ITGB2, CCL5, CXCL12 | 9.73E-04 |
| KEGG_PATHWAY | hsa04610:Complement and coagulation cascades | C1QB, C3, TFPI, SERPING1, SERPINA1, C1S | 0.001993784 |
| KEGG_PATHWAY | hsa05142:Chagas disease (American trypanosomiasis) | TNFRSF1A, C1QB, CCL2, GNAQ, C3, TLR2, CCL5 | 0.002305064 |
| KEGG_PATHWAY | hsa05133:Pertussis | C1QB, C3, SERPING1, ITGB2, C1S, ITGAM | 0.002879817 |
